# Information-theoretic and physical constraints on advanced neural signal decoding

**DOI:** 10.3389/fnsys.2026.1786729

**Published:** 2026-04-28

**Authors:** Enzhuo Zhang, Syed Ishtiaque Ahmed

**Affiliations:** 1 University of Toronto Scarborough, Toronto, ON, Canada; 2 Department of Computer Science, University of Toronto St George, Toronto, ON, Canada

**Keywords:** information theory, neural signal decoding, physical constraints, quantum-inspired frameworks, theoretical neuroscience

## Abstract

Recent interdisciplinary research has raised interest in whether non-classical physical principles may impose fundamental constraints on how neural information can be observed, extracted, or decoded. While conventional neuroscience models neural signaling primarily through classical electrochemical processes, a growing body of theoretical literature has speculated that quantum-mechanical concepts—such as coherence, entanglement, or quantum-inspired information processing—may offer alternative perspectives on the limits of neural observability. This article provides a critical and integrative review of theoretical proposals situated at the intersection of neuroscience, quantum biology, and information theory, without assuming the physical realizability of quantum information processing in biological neural systems. We examine conceptual motivations, key physical constraints (including decoherence, thermal noise, and system complexity), and unresolved theoretical challenges that arise when extending classical neural decoding frameworks toward non-classical regimes. Rather than proposing an experimentally validated mechanism for brain decoding, this work focuses on identifying conceptual boundaries, potential misinterpretations, and open questions that must be addressed before non-classical approaches to neural signal decoding can be meaningfully evaluated. Ethical considerations, methodological limitations, and future research directions are discussed to clarify the conditions under which such speculative frameworks may contribute to neuroscience, while avoiding overextension beyond current empirical evidence.

## 1 Introduction

Contemporary neuroscience has largely explained neural signaling, cognition, and behavior through classical electrochemical and network-based models. Within this framework, neural activity is understood as arising from ion channel dynamics, synaptic transmission, and large-scale interactions among distributed neuronal populations. These classical approaches have proven highly successful in accounting for a wide range of experimental observations and remain the dominant explanatory paradigm in neuroscience.

In parallel, a smaller body of interdisciplinary theoretical literature has speculatively explored whether non-classical physical principles, drawn from quantum mechanics and quantum biology, might impose additional constraints or offer alternative perspectives on neural information processing. Motivated by advances in quantum physics, quantum information theory, and the observation of quantum effects in certain biological systems, these discussions have raised questions about whether classical descriptions fully capture the limits of neural observability, integration, and information encoding (Marais et al., 2018; Lambert et al., 2013; Arndt et al., 2009; Qian and Beltran, 2022; Mokhtari et al., 2024). It is important to emphasize that such ideas remain controversial and are not part of mainstream neuroscience, yet they continue to generate conceptual debate across physics, biology, and cognitive science (Marais et al., 2018; Hameroff and Roger, 2014).

One strand of this literature adopts *quantum-inspired* frameworks to model cognitive phenomena such as decision-making, uncertainty, and contextual dependence. In these approaches, mathematical formalisms originating from quantum theory—such as superposition, interference, and contextual measurement—are employed as descriptive tools rather than as claims about underlying physical quantum processes occurring in neural tissue. Quantum cognitive models have been developed and applied in psychology and computational neuroscience as alternative representational frameworks, while remaining agnostic regarding their biological implementation (Busemeyer and Bruza, 2012).

Another strand, rooted in quantum biology, investigates whether genuine quantum phenomena—such as coherence, tunneling, or spin dynamics—can persist in biological environments despite thermal noise and decoherence. Empirical evidence from photosynthetic complexes, enzymatic reactions, and other molecular systems has demonstrated that quantum effects can play functional roles under specific biological conditions (Lambert et al., 2013; Arndt et al., 2009; McFadden, 2014; Nunn et al., 2016). These findings have motivated speculation about whether analogous mechanisms could, in principle, influence neural processes. However, extending such results to the brain introduces substantial theoretical challenges, given its warm, noisy, and highly complex environment (Tegmark, 2000; Fisher, 2015).

Within this context, more ambitious hypotheses have proposed that quantum coherence or entanglement might play a functional role in neural information processing or even consciousness. Notably, the orchestrated objective reduction (Orch OR) model suggested that quantum processes within neuronal microtubules could contribute to conscious experience (Hameroff and Roger, 2014). While such proposals have stimulated interdisciplinary discussion, they have also attracted substantial criticism. Quantitative analyses have argued that quantum states in neural systems would decohere on timescales far shorter than those relevant for neuronal signaling, thereby challenging the biological plausibility of such mechanisms (Tegmark, 2000). As a result, the distinction between quantum-inspired metaphors, speculative physical hypotheses, and experimentally grounded mechanisms remains a central point of contention in this field (Jedlicka, 2017).

Against this backdrop, the concept sometimes referred to as “quantum brain reading” has emerged as a broad and loosely defined collection of ideas concerning whether non-classical physical principles might affect how neural information could, in theory, be accessed, interpreted, or constrained. In much of the existing discourse, the term has been used inconsistently, occasionally blurring the boundary between conceptual exploration and claims of technological feasibility. This ambiguity has contributed to conceptual overextension and to ethical concerns surrounding brain recording and mind-reading technologies (Rainey et al., 2020).

The aim of this article is not to propose or validate an experimentally realizable method for decoding brain activity using quantum-mechanical processes. Instead, this work provides a critical and integrative review of theoretical proposals that invoke non-classical physical concepts in discussions of neural information decoding. By examining physical constraints such as decoherence, thermal noise, and biological complexity (Tegmark, 2000; Swan et al., 2022; Matarèse et al., 2023), alongside information-theoretic considerations and ethical implications (Rainey et al., 2020), this paper seeks to clarify the conceptual boundaries of these approaches. In doing so, it aims to distinguish speculative thought experiments and quantum-inspired interpretations from physically grounded mechanisms, and to identify the conditions under which such ideas may—or may not—meaningfully inform future interdisciplinary research.

## 2 Related work

### 2.1 Quantum-inspired models of cognition and information processing

A distinct line of research applies mathematical formalisms derived from quantum theory as *conceptual tools* for modeling cognitive phenomena, without assuming the presence of physical quantum processes in neural tissue. In this literature, principles such as superposition, contextuality, and probabilistic interference are used to describe decision-making, uncertainty, and information integration in psychological processes (Busemeyer and Bruza, 2012). These quantum-inspired cognitive models reinterpret cognitive states within non-classical probability frameworks, offering alternatives to classical Bayesian or Boolean representations.

Importantly, proponents of quantum cognition explicitly distinguish their approach from claims about quantum-mechanical implementation in the brain. The use of quantum probability theory in this context is formal rather than physical, and no assertion is made that neurons or synapses function as quantum systems (Busemeyer and Bruza, 2012). As such, quantum-inspired cognitive models are best understood as representational frameworks that highlight limitations of classical modeling approaches, rather than as evidence for quantum neurobiology.

Related developments in quantum-inspired computation and machine learning have further influenced discussions of information processing in complex systems. These approaches explore how non-classical mathematical structures can be leveraged for optimization, pattern recognition, and associative memory tasks (Schuld et al., 2014). While such models are not biologically instantiated, they contribute to broader debates regarding the theoretical limits of classical computation and the potential value of alternative information-processing paradigms.

### 2.2 Quantum biology and molecular-scale evidence

Parallel to developments in quantum-inspired cognition, the field of quantum biology has produced empirical evidence that quantum effects can play functional roles in certain biological systems. Experimental and theoretical studies have demonstrated the presence of quantum coherence, tunneling, and wave-like energy transport in photosynthetic complexes, enzymatic reactions, and molecular transport processes (Lambert et al., 2013; Arndt et al., 2009). These findings challenge the assumption that quantum phenomena are universally suppressed in warm, noisy biological environments.

Subsequent reviews have proposed that biological organization, environmental coupling, and dynamical regulation may enable transient quantum effects at the molecular scale (Marais et al., 2018; Swan et al., 2022). However, these effects are typically short-lived and spatially localized, and their functional relevance is confined to specific biochemical contexts. It is also important to note that quantum coherence can manifest at macroscopic scales under certain physical conditions. Phenomena such as superconductivity and Bose–Einstein condensation demonstrate that collective quantum states can persist across large ensembles of particles. Similarly, concepts such as Anderson localization illustrate that quantum behavior may remain robust in disordered environments. While these phenomena arise in highly specific physical systems that differ fundamentally from neural tissue, they highlight that quantum effects are not universally restricted to microscopic scales (Arndt et al., 2009; Marais et al., 2018; Cordier et al., 2022). Extending such mechanisms to neural systems introduces additional constraints related to system size, thermal noise, structural heterogeneity, and environmental coupling (Tegmark, 2000; Marais et al., 2018).

To date, no empirical evidence supports the existence of biologically functional quantum coherence or entanglement operating at the spatial and temporal scales required for neural information processing. As a result, most authors emphasize that molecular-scale quantum phenomena cannot be straightforwardly extrapolated to macroscopic neural networks (Tegmark, 2000; Jedlicka, 2017).

### 2.3 Conceptual distinctions: quantum coherence vs. entanglement

Discussions of non-classical effects in biological or neural systems often conflate the concepts of quantum coherence and quantum entanglement, despite their distinct physical meanings and feasibility requirements. Quantum coherence refers to the maintenance of well-defined phase relationships within a single quantum system, enabling superposition and wave-like behavior. In contrast, quantum entanglement describes non-classical correlations between multiple subsystems, such that the state of one cannot be described independently of the others.

From a physical standpoint, coherence is generally more robust than entanglement in noisy environments, as it does not require the preservation of non-local correlations across multiple degrees of freedom (Lambert et al., 2013; Swan et al., 2022). Even so, coherence in biological systems is typically transient and limited to specific molecular contexts. Entanglement, by contrast, is substantially more fragile and susceptible to decoherence arising from thermal fluctuations and environmental interactions (Matarèse et al., 2023).

In the context of neuroscience, this distinction is critical. While quantum coherence has been empirically demonstrated in certain biological molecules, no evidence supports the presence of sustained entanglement in neural systems. Consequently, references to entanglement in discussions of brain function are best interpreted as metaphorical or as theoretical boundary cases rather than as physically established mechanisms (Jedlicka, 2017). Failure to maintain this distinction has contributed to conceptual ambiguity and skepticism within the neuroscience community.

### 2.4 Summary of controversies and theoretical limits

Collectively, prior work at the intersection of neuroscience, quantum theory, and information science highlights both the appeal and the limitations of non-classical perspectives on brain function. Quantum-inspired cognitive models provide useful alternative formalisms without making claims about physical implementation (Busemeyer and Bruza, 2012). Quantum biology demonstrates that quantum effects can be biologically relevant under narrowly defined molecular conditions, yet these findings do not generalize to neural systems (Lambert et al., 2013; Marais et al., 2018). Meanwhile, speculative extensions invoking coherence or entanglement at the level of neural networks remain controversial and lack empirical support (Jedlicka, 2017).

These strands of literature underscore the importance of clearly distinguishing conceptual models, metaphorical interpretations, and physically grounded mechanisms. Related work thus provides no evidence for non-classical neural decoding, but rather a set of theoretical constraints and cautionary lessons that frame contemporary discussions of neural observability and information processing.

## 3 Challenges in non-classical approaches to neural signal decoding

### 3.1 Classical neurophysiological constraints

Any discussion of non-classical approaches to neural signal decoding must begin with the well-established physical and biological properties of neural tissue. Neural information processing in the brain is fundamentally governed by classical electrochemical mechanisms, including ion-channel dynamics, membrane potentials, and synaptic transmission. These processes operate in a warm, aqueous, and chemically active environment characterized by continuous thermal motion, stochastic fluctuations, and strong coupling to surrounding biological matter.

From a physical perspective, such conditions impose severe constraints on the persistence of delicate quantum states. Thermal noise, molecular collisions, and electromagnetic interactions continuously perturb neural components, leading to rapid loss of phase coherence in any putative quantum degrees of freedom (Tegmark, 2000; Jedlicka, 2017). Unlike engineered quantum systems, which require extreme isolation and cryogenic temperatures to suppress environmental interactions, neural tissue lacks mechanisms for shielding or error correction at the level required to preserve quantum coherence or entanglement.

**Figure 1:**
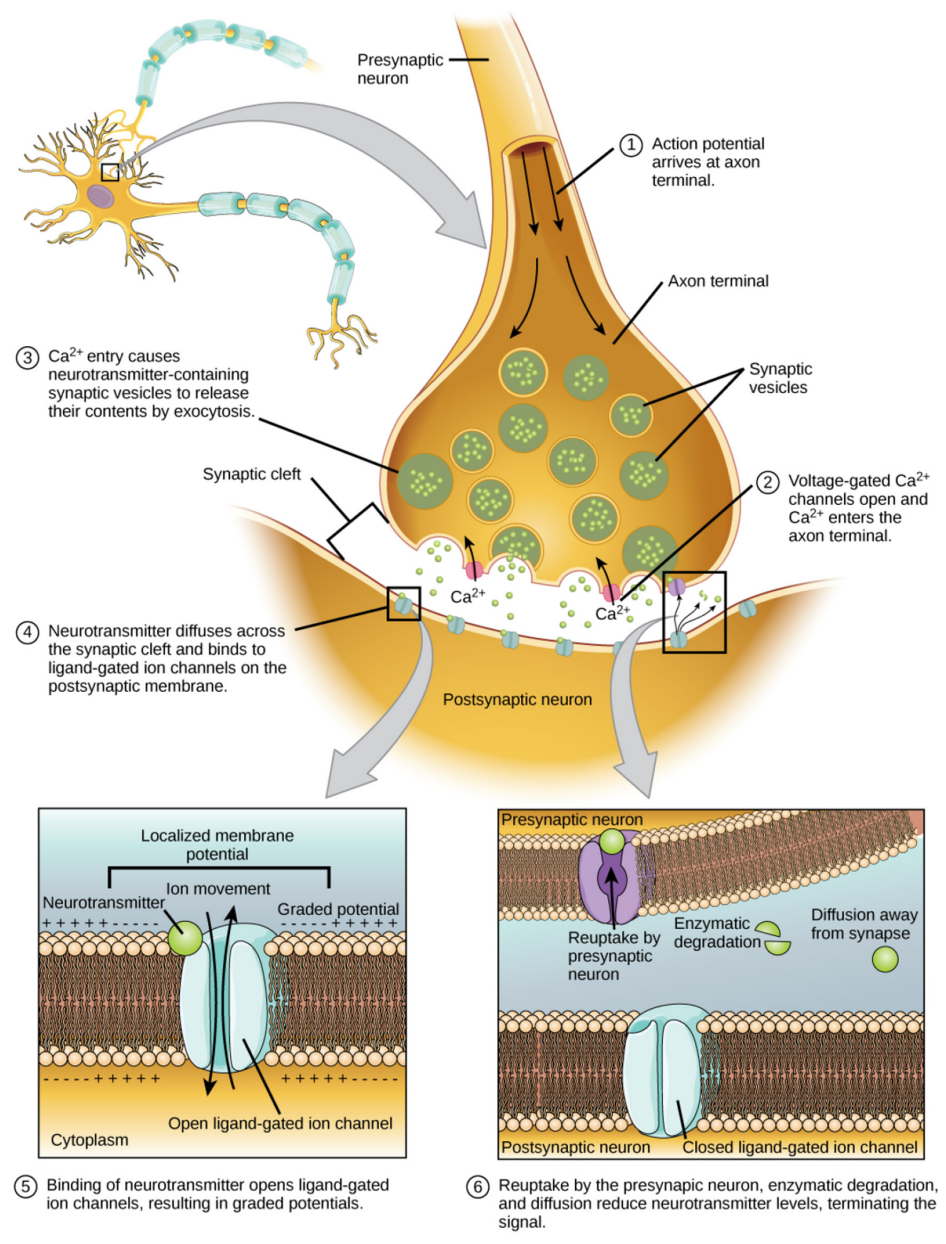
Classical electrochemical mechanisms underlying neural signal transmission. Action potential generation and propagation depend on ion fluxes across neuronal membranes, driven by thermal motion and stochastic channel dynamics. This well-established classical signaling architecture highlights the warm, noisy biological environment in which neural information processing occurs, illustrating the severe physical constraints faced by any proposal invoking sustained quantum coherence or entanglement in neural systems. Adapted from Douglas College (2018).

As illustrated in Figure 1, neural signaling relies on the controlled but inherently stochastic flux of ions across neuronal membranes, driven by electrochemical gradients and thermal energy.

Action potential generation and propagation depend on large numbers of ion-channel opening and closing events, each subject to probabilistic behavior and environmental noise. This classical signaling architecture highlights the physical context in which neural information processing occurs and underscores the difficulty of maintaining non-classical quantum states within neural systems. The inclusion of Figure 1 is therefore intended not as background instruction, but as an explicit illustration of the biological environment that constrains proposals invoking sustained quantum coherence or entanglement in the brain.

### 3.2 Scale, complexity, and the difficulty of forming non-classical correlations in the brain

**Figure 2:**
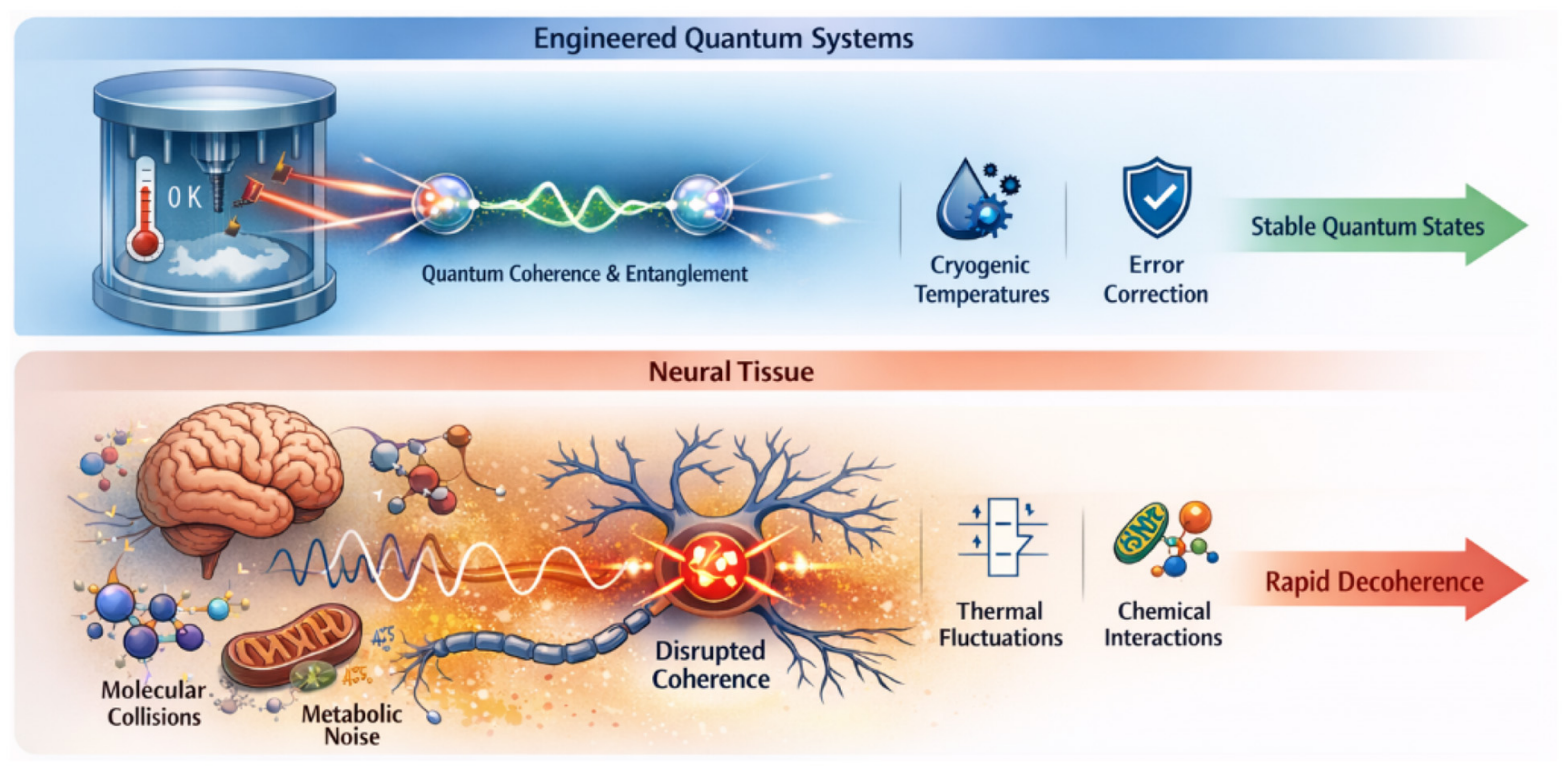
Environmental constraints on quantum coherence and entanglement in neural systems. Conceptual illustration of how environmental interactions, thermal noise, and biological complexity influence the stability of non-classical correlations. In engineered quantum systems, coherence and entanglement are typically maintained through strong isolation, cryogenic conditions, or active error correction. In contrast, neural tissue operates in a warm, chemically active, and highly interactive environment, where molecular collisions, electromagnetic fluctuations, and metabolic processes continuously perturb physical states. These factors lead to rapid decoherence, posing significant challenges for sustaining stable quantum correlations at biologically relevant spatial and temporal scales. Conceptual illustration created by the authors with the assistance of AI-based visualization tools. The authors take full responsibility for the scientific accuracy of the figure.

Beyond the classical electrochemical constraints discussed above, a central challenge for non-classical approaches to neural signal decoding concerns the formation and maintenance of quantum coherence or entanglement within neural systems. From a physical standpoint, quantum entanglement represents a particularly stringent form of non-classical correlation, requiring precise isolation from environmental interactions and tight control over system dynamics. While many engineered quantum systems rely on strong isolation to preserve entanglement, recent studies have shown that non-classical correlations can also emerge in open systems through mediating interactions such as phonons or collective excitations. In certain physical contexts, entanglement may display unexpected robustness against environmental disorder, although maintaining such states in complex biological environments remains highly challenging (Cordier et al., 2022; Matarèse et al., 2023). Such requirements stand in stark contrast to the highly interactive, thermally noisy conditions characteristic of biological neural tissue (Tegmark, 2000). These constraints are conceptually summarized in Figure 2.

Biological environments introduce multiple sources of decoherence that act simultaneously across spatial and temporal scales. In neural systems, molecular collisions, fluctuating electromagnetic fields, synaptic activity, and metabolic processes continuously perturb local physical states. These interactions rapidly destroy phase relationships and non-local correlations, rendering sustained entanglement exceedingly difficult to preserve under physiological conditions (Tegmark, 2000; Swan et al., 2022; Matarèse et al., 2023). Unlike engineered quantum systems, which rely on cryogenic temperatures and active error correction to maintain coherence, neural tissue lacks known mechanisms capable of providing comparable protection. Recent experimental work has further demonstrated that certain engineered quantum systems can sustain coherence at or near room temperature. Molecular spin systems and nitrogen-vacancy (NV) centers in diamond have shown that quantum coherence and spin-based quantum states can persist under ambient or near-room-temperature conditions in relatively noisy environments under specific conditions. These findings suggest that the relationship between temperature, environmental complexity, and quantum coherence is more nuanced than previously assumed. Nevertheless, translating such mechanisms to neural systems remains speculative due to differences in scale, structure, and biological dynamics (Nishikawa et al., 2025).

A further complication arises from the scale mismatch between known quantum biological phenomena and neural information processing. Empirically observed quantum effects in biological systems are typically confined to molecular or sub-molecular structures and operate over nanometer spatial scales and femtosecond timescales (Marais et al., 2018; Lambert et al., 2013). Neural computation, by contrast, emerges from the coordinated activity of large populations of neurons distributed across macroscopic spatial scales. Bridging this gap would require mechanisms capable of amplifying or integrating microscopic quantum effects into stable, system-level neural dynamics without rapid decoherence—an outcome for which no experimentally supported model currently exists (Tegmark, 2000; Jedlicka, 2017).

In addition, proposals invoking entanglement in neural systems often underestimate the challenges associated with system identification and control. Entanglement requires well-defined subsystem boundaries and controlled interactions, yet neural systems are characterized by continuous coupling, heterogeneity, and dynamic reconfiguration. The absence of clear physical boundaries between relevant degrees of freedom complicates both the definition and detection of entangled states in biological contexts (Matarèse et al., 2023). As a result, claims regarding neural entanglement frequently rely on metaphorical interpretations rather than physically verifiable correlations (Jedlicka, 2017).

Taken together, these considerations suggest that forming biologically functional quantum entanglement within the brain faces severe and currently unresolved physical obstacles. While entanglement may serve as a useful theoretical boundary condition for exploring the limits of non-classical information processing, existing evidence does not support its realization in neural systems at scales relevant for cognition or signal decoding (Tegmark, 2000; Swan et al., 2022; Jedlicka, 2017). Recognizing these limitations is essential for maintaining conceptual clarity and for preventing overextension in discussions of quantum approaches to neuroscience.

### 3.3 Measurement, observability, and information-theoretic limits

Even if non-classical states were hypothetically present within neural systems, a further set of challenges arises from the problem of measurement and observability. Neural activity is inherently high-dimensional, stochastic, and distributed across multiple spatial and temporal scales. Any attempt to extract fine-grained information from such systems—whether using classical or speculative non-classical approaches—must contend with substantial background noise, redundancy, and uncertainty.

From a physical perspective, measurement in biological systems is intrinsically invasive. Neural signals are embedded within dense electrochemical activity, and attempts to probe microscopic states inevitably introduce perturbations that alter the system being observed. This issue is not unique to quantum frameworks; however, it becomes particularly acute when considering non-classical states, which are highly sensitive to environmental interactions and measurement back-action (Marais et al., 2018; Tegmark, 2000). As a result, even the act of observation can rapidly destroy the very correlations one seeks to detect.

Information-theoretic considerations further constrain neural signal decoding. Limits on signal-to-noise ratios, channel capacity, and measurement precision impose fundamental bounds on how much information can be reliably extracted from neural systems (Cordier et al., 2022; Weidner et al., 2023). These constraints apply regardless of whether decoding strategies are framed in classical or quantum-inspired terms. In practice, neural activity exhibits substantial variability across trials and individuals, further reducing the feasibility of isolating stable informational variables at fine temporal or spatial resolutions.

Another challenge concerns the interface between putative non-classical descriptions and classical neural dynamics. Neural computation ultimately manifests through macroscopic variables such as membrane potentials, firing rates, and network-level patterns. Translating any hypothesized non-classical state into observable classical signals would require a well-defined mapping between quantum-level descriptions and classical readouts. To date, no experimentally supported framework has demonstrated how such a quantum–classical interface could operate in biologically realistic neural systems (Swan et al., 2022; Jedlicka, 2017).

Finally, advances in sensing technologies—including quantum-enhanced magnetometry and other high-sensitivity measurement devices—are sometimes cited as potential tools for improving neural observability. While such technologies have achieved remarkable precision in controlled physical settings, their relevance to neural systems remains limited. Importantly, the use of quantum-enhanced sensors does not imply that neural processes themselves are quantum-mechanical; rather, it reflects improvements in measurement instrumentation (Cordier et al., 2022; Vettoliere and Granata, 2023). Consequently, enhanced sensitivity alone does not overcome the fundamental biological and information-theoretic constraints discussed above.

Taken together, these measurement and observability challenges suggest that non-classical approaches to neural signal decoding face severe practical limitations even under optimistic assumptions. The difficulty lies not only in whether non-classical states could exist in neural systems, but also in whether meaningful information could be extracted from such states without disrupting the underlying biological processes. Recognizing these constraints is essential for distinguishing speculative theoretical exploration from physically and informationally grounded neuroscience.

Emerging neuromorphic computing systems may also offer experimental platforms for exploring theoretical questions related to neural information processing. Artificial neurons implemented through memristive devices or hybrid computing architectures can reproduce certain statistical properties of biological neurons while remaining experimentally controllable. Such systems may provide a bridge between theoretical models and experimentally testable frameworks, allowing researchers to investigate quantum-inspired computational principles without requiring quantum states within biological neural tissue (Schuld et al., 2014; Kumar et al., 2017; Cordier et al., 2022).

### 3.4 Conceptual ambiguity and ethical constraints

A persistent challenge in discussions of non-classical approaches to neural signal decoding lies in conceptual ambiguity. Quantum concepts such as superposition, coherence, entanglement, and quantum information are often employed across different levels of description—mathematical, metaphorical, and physical—without clear delineation. In some cases, formal tools derived from quantum theory are used to model cognitive or informational phenomena, while in others similar terminology is invoked to suggest underlying physical mechanisms in neural tissue. This lack of terminological precision has contributed to confusion and skepticism within the neuroscience community (Jedlicka, 2017).

Failure to distinguish between *quantum-inspired* models and claims of *quantum-mechanical implementation* is particularly problematic. Quantum-inspired frameworks may offer useful mathematical or conceptual perspectives without implying that neural systems operate according to quantum dynamics at a physical level (Busemeyer and Bruza, 2012). When these distinctions are not explicitly stated, speculative interpretations risk being misread as assertions of biological feasibility, leading to conceptual overextension and miscommunication across disciplinary boundaries (Jedlicka, 2017).

Relatedly, the term “brain reading” itself is often used imprecisely, encompassing a wide range of techniques that differ substantially in scope, resolution, and feasibility. Classical neural decoding methods, such as electrophysiological recording and neuroimaging, operate at coarse spatial and temporal scales and are subject to well-characterized limitations. Speculative extensions invoking non-classical principles must therefore be evaluated with particular care to avoid conflating metaphorical interpretation with technological capability. Maintaining conceptual clarity is essential for ensuring that theoretical exploration does not inadvertently promote unrealistic expectations regarding neural observability.

Ethical considerations further constrain discussions of advanced neural decoding. Even within established classical frameworks, brain-recording technologies raise concerns related to privacy, autonomy, informed consent, and the potential misuse of neural data (Rainey et al., 2020). These concerns are amplified when speculative or future-oriented frameworks are presented without explicit acknowledgment of their uncertainty or limitations. Public misunderstanding of speculative claims may contribute to exaggerated perceptions of technological capability, thereby intensifying ethical and societal risks.

Responsible scholarship in this area therefore requires transparency regarding the status of proposed ideas, including whether they are intended as metaphorical models, theoretical boundary analyses, or experimentally grounded mechanisms. Ethical discourse should not be treated as an afterthought, but rather as an integral component of theoretical evaluation. Explicitly articulating conceptual boundaries and feasibility constraints helps mitigate the risk of overinterpretation and supports more constructive interdisciplinary dialogue (Rainey et al., 2020).

Taken together, conceptual ambiguity and ethical considerations reinforce the need for caution in extending non-classical frameworks to neural signal decoding. Addressing these challenges does not preclude speculative or interdisciplinary inquiry; rather, it ensures that such inquiry remains grounded, interpretable, and socially responsible. Clear distinctions between theory, metaphor, and mechanism are essential for advancing understanding while avoiding conceptual and ethical overreach.

## 4 Discussion and outlook

The preceding sections have examined non-classical approaches to neural signal decoding through the lenses of prior literature, physical constraints, conceptual frameworks, and ethical considerations. Taken together, these analyses indicate that proposals invoking quantum coherence or entanglement in neural systems face severe and currently unresolved challenges. These challenges arise not from a lack of theoretical creativity, but from well-established properties of neural tissue, biological noise, scale mismatch, measurement limitations, and information-theoretic bounds.

**Figure 3:**
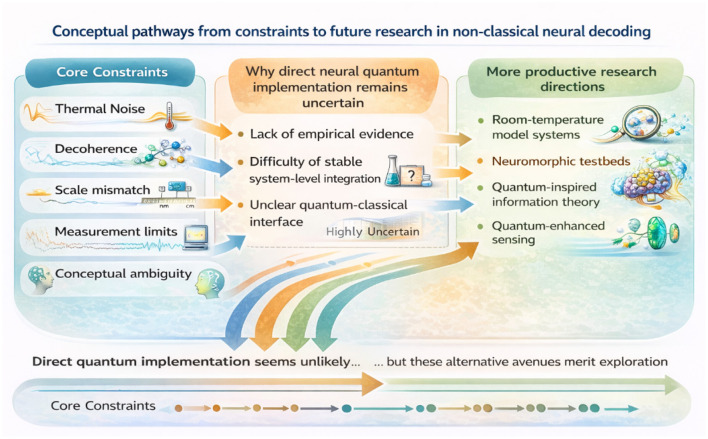
Conceptual pathways from physical constraints to future research directions in non-classical neural decoding. Schematic summary of how major physical, biological, and conceptual constraints—including thermal noise, decoherence, scale mismatch, measurement limitations, and conceptual ambiguity—shape current interpretations of non-classical approaches to neural signal decoding. These constraints collectively limit the feasibility of direct quantum implementation in neural systems, highlighting challenges such as the lack of empirical evidence, difficulty of stable system-level integration, and the unclear interface between quantum and classical descriptions. Rather than supporting direct quantum effects in neural tissue, these limitations motivate alternative research directions, including room-temperature model systems, neuromorphic platforms, quantum-inspired theoretical frameworks, and quantum-enhanced sensing technologies. Conceptual illustration created by the authors with the assistance of AI-based visualization tools. The authors take full responsibility for the scientific accuracy of the figure.

Importantly, the central conclusion of this review is not that non-classical perspectives are categorically irrelevant to neuroscience. These conceptual relationships are summarized in Figure 3.

Rather, the analysis suggests that such perspectives are most appropriately interpreted as tools for exploring *theoretical limits* and *conceptual boundaries*, rather than as candidates for near-term or physically realizable neural decoding technologies. Distinguishing between speculative boundary analyses and claims of mechanistic implementation is essential for maintaining both scientific rigor and interdisciplinary credibility.

From a conceptual standpoint, quantum-inspired frameworks have demonstrated value as alternative mathematical or representational tools for modeling aspects of cognition and information processing. These approaches highlight limitations of classical probabilistic and computational models without requiring physical quantum dynamics in neural tissue. Similarly, insights from quantum biology underscore that quantum effects can play functional roles in select biological systems under narrowly defined conditions, while simultaneously illustrating how difficult it is to generalize such effects to macroscopic, noisy neural environments.

The discussion of physical constraints—including decoherence, environmental coupling, and system complexity—further clarifies why proposals for non-classical neural decoding must be approached with caution. Even under optimistic assumptions, the formation, preservation, and measurement of biologically functional non-classical states in the brain remain unsupported by empirical evidence. Moreover, information-theoretic considerations suggest that any potential advantage conferred by quantum-inspired measurement paradigms would be severely limited by noise, redundancy, and observability constraints inherent to neural systems.

Future research in this interdisciplinary domain may benefit from several promising directions that bridge neuroscience, quantum physics, and information science. One potential avenue involves the development of theoretical frameworks that explore the limits of classical neural information processing while incorporating insights from quantum information theory. Such approaches may help clarify whether certain informational constraints observed in neural systems arise purely from classical complexity or from deeper physical limits.

Another promising direction lies in the investigation of room-temperature quantum systems that exhibit partial coherence under noisy conditions. Recent studies of molecular spins, nitrogen-vacancy (NV) centers in diamond, and related quantum materials suggest that certain quantum states can persist in complex environments without cryogenic isolation. While these systems are not biological, they may serve as experimental analogs for exploring how non-classical correlations behave in thermally active environments.

In addition, neuromorphic computing and artificial neural systems may provide controllable platforms for testing theoretical hypotheses concerning quantum-inspired information processing. Artificial neurons implemented through memristive networks or hybrid classical–quantum architectures could allow researchers to experimentally evaluate how quantum-inspired computational principles influence learning, pattern recognition, or information integration.

Finally, advances in quantum sensing technologies may continue to improve the sensitivity of neural measurement techniques. Although such devices do not imply that neural processes themselves are quantum mechanical, they may nonetheless contribute to more precise observations of neural dynamics and help refine theoretical models of neural information limits.

**Figure 4:**
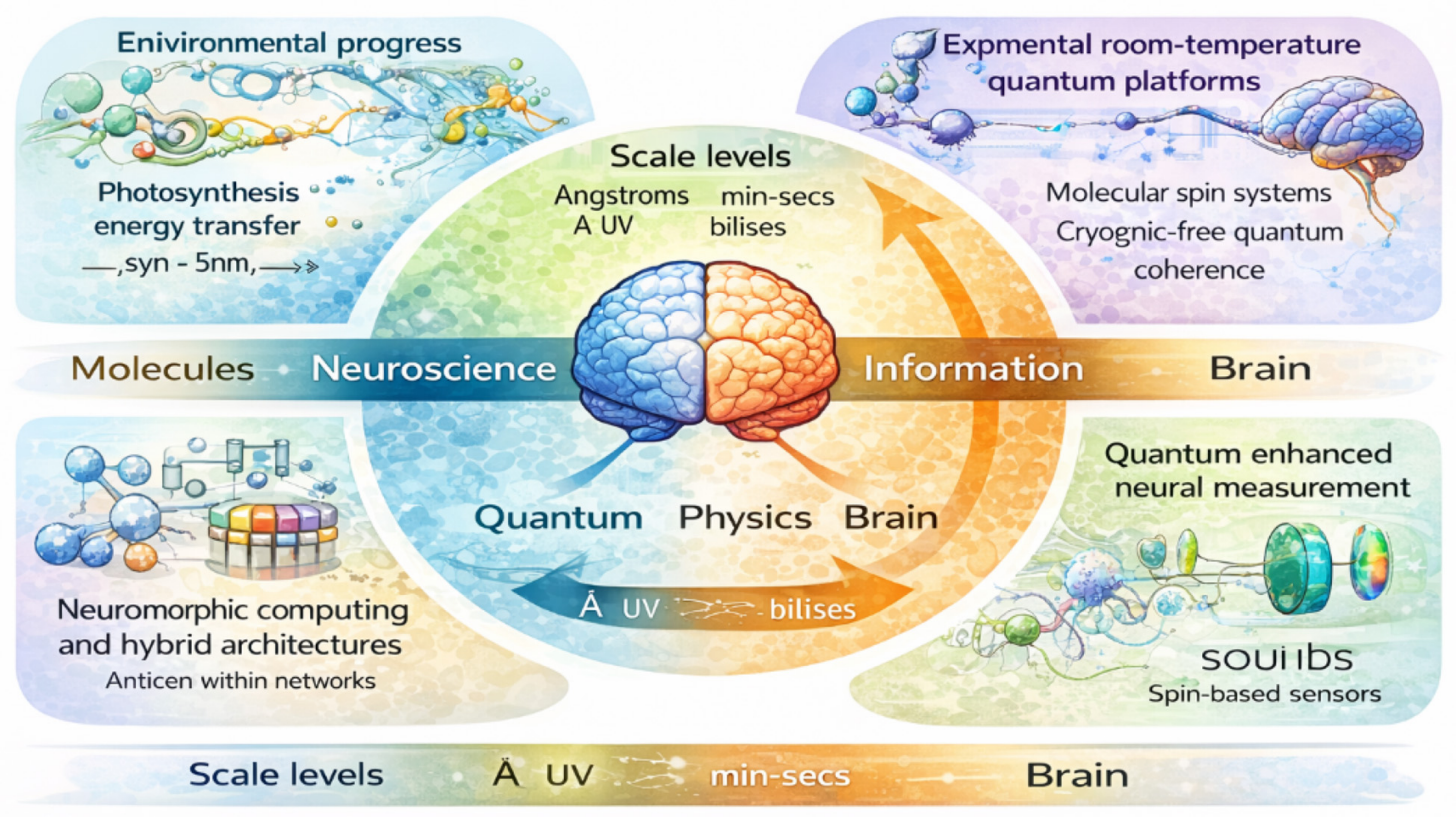
Potential interdisciplinary research directions linking neuroscience, quantum physics, and information theory. Conceptual illustration of emerging research pathways at the intersection of neuroscience, quantum science, and information theory. These include theoretical investigations of neural information limits, room-temperature quantum platforms, neuromorphic computing architectures, and quantum-enhanced sensing technologies. Together, these directions highlight possible avenues for exploring non-classical principles in neural systems without assuming direct quantum implementation in biological tissue. Created by the authors with the assistance of AI-based visualization tools. The authors take full responsibility for the scientific accuracy of the figure.

Looking forward, future interdisciplinary research may benefit from reframing questions about non-classical neural processes away from feasibility-driven narratives and toward boundary-oriented inquiry. These potential interdisciplinary research directions are conceptually illustrated in Figure 4. Rather than asking whether the brain “uses” quantum mechanics in a functional sense, it may be more productive to ask what classical descriptions omit, where their limits lie, and how alternative formalisms can sharpen our understanding of neural observability and information integration. Importantly, such theoretical models need not be strictly confined to microscopic descriptions. Macroscopic or mesoscopic frameworks incorporating collective dynamics may also provide meaningful insights into how non-classical information principles could interact with neural systems. In this context, quantum concepts can serve as rigorous theoretical reference points, even if they are not physically instantiated in neural tissue.

Advances in sensing and measurement technologies may continue to improve the resolution with which neural activity can be observed (Qi and Kang, 2023). However, it is essential to emphasize that improvements in measurement instrumentation—whether quantum-enhanced or otherwise—do not imply corresponding changes in the underlying physical nature of neural processes. Future work should therefore maintain a clear distinction between the properties of measurement devices and the properties of the systems being measured.

Finally, ethical considerations must remain integral to discussions of advanced neural decoding. As speculative ideas circulate beyond academic contexts, the risk of misinterpretation and overstatement increases. Responsible scholarship requires explicit communication of uncertainty, limitations, and feasibility constraints to prevent unrealistic expectations and to safeguard public trust. Integrating ethical reflection with theoretical analysis is not a limitation on scientific inquiry, but a necessary condition for its responsible advancement.

In conclusion, non-classical approaches to neural signal decoding currently function best as conceptual frameworks for probing the limits of existing models rather than as foundations for actionable technologies. By clarifying physical constraints, conceptual distinctions, and ethical boundaries, this review aims to contribute to a more disciplined and productive dialogue at the intersection of neuroscience, physics, and information theory. Such clarity is essential if interdisciplinary exploration is to advance understanding without sacrificing rigor or responsibility.

## Data Availability

The original contributions presented in the study are included in the article/supplementary material, further inquiries can be directed to the corresponding authors.
